# SSR Locator: Tool for Simple Sequence Repeat Discovery Integrated with Primer Design and PCR Simulation

**DOI:** 10.1155/2008/412696

**Published:** 2008-07-27

**Authors:** Luciano Carlos da Maia, Dario Abel Palmieri, Velci Queiroz de Souza, Mauricio Marini Kopp, Fernando Irajá Félix de Carvalho, Antonio Costa de Oliveira

**Affiliations:** ^1^Plant Genomics and Breeding Laboratory, Eliseu Maciel School of Agronomy, Federal University of Pelotas, Pelotas, RS 96.001-970, Brazil; ^2^Laboratory for Environmental Studies, Catholic University of Salvador, Salvador, BA, 40.220-140, Brazil

## Abstract

Microsatellites or SSRs (*simple sequence repeats*) are ubiquitous short tandem duplications occurring in eukaryotic organisms. These sequences are among the best marker technologies applied in plant genetics and breeding. The abundant genomic, BAC, and EST sequences available in databases allow the survey regarding presence and location of SSR loci. Additional information concerning primer sequences is also the target of plant geneticists and breeders. In this paper, we describe a utility that integrates SSR searches, frequency of occurrence of motifs and arrangements, primer design, and PCR simulation against other databases. This simulation allows the performance of global alignments and identity and homology searches between different amplified sequences, that is, amplicons. In order to validate the tool functions, SSR discovery searches were performed in a database containing 28 469 nonredundant rice cDNA sequences.

## 1. INTRODUCTION

Microsatellites or SSRs (*simple sequence repeats*) are sequences in which one or few bases are tandemly repeated for varying numbers of times [1]. Variations in SSR regions originate mostly from errors during the replication process, frequently DNA polymerase slippage, generating insertion or deletion of base pairs, resulting, respectively, in larger or smaller regions [2, 3]. SSR assessments in the human genome have shown that many diseases are caused by mutation in these sequences [4].

SSRs can be found in different regions of genes, that is, coding sequences, untranslated sequences (5′-UTR and 3′-UTR), and introns, where the expansions and/or contractions can lead to gene gain or loss of function [5]. Also, there are evidences that genomic distribution of SSRs is related to chromatin organization, recombination, and DNA repair. SSRs are found throughout the genome, in both protein-coding and noncoding regions. Genome fractions as low as 0.85% (*Arabidopsis thaliana*), 0.37% (*Zea mays*), 0.21% (*Caenorhabtis elegans*), 0.30% (*Sacharomyces cerevisae*) and as high as 3.0% (*Homo sapiens*) and 3.21% (*Fugu rubripes*) have been found. Some bias for defined genomic locations has also been reported [6, 7]. This class of markers is broadly applied in genetics and plant breeding, due to its reproducibility, multiallelic, codominant nature, and genomic abundance. Its use for integrating genetic maps, physical mapping, and anchoring gives geneticists and plant breeders a pathway to link genotype and phenotype variations [8].

The protocols for isolating SSR loci for a new species were always very labor-intensive. Currently, with the accumulation of biological data originating from whole genome sequence initiatives, the use of bioinformatics tools helps to maximize the identification of these sequences and consequently, the efficiency in the number of generated markers [9].

The first in silico studies of SSRs were developed using FASTA [10] and BLAST [11] packages. Later, more specific algorithms, such as SPUTINICK [12], REPEATMASKER [13], TRF-*Tandem Repeat Find* [14], TROLL [15], MISA [16] and SSRIT (*Simple Sequence Repeat Tool*) [17], were obtained [9]. 

SSR detection is generally followed by the use of another program for primer design, to be anchored on flanking sequences. Also, in some applications, a third step using e-PCR [18] is added, with the goal of verifying primer redundancy. The sequential use of a number of software is often called a pipeline. Building such a pipeline can be a very difficult task for research groups not familiar with programming tools.

In the present work, a computing tool with an interface for Windowsusers was developed, called SSR Locator. The application integrates the following functions: (i) detection and characterization of SSRs and minisatellite motifs between 1 and 10 base pairs; (ii) primer design for each locus found; (iii) simulation of PCR (polymerase chain reaction), amplifying fragments with different primer pairs from a given set of fasta files; (iv) global alignment between amplicons generated by the same primer pair; and (v) estimation of global alignment scores and identities between amplicons, generating information on primer specificity and redundancy. The described tool is publicly available at the site http://www.ufpel.edu.br/~lmaia.faem.

## 2. MATERIAL AND METHODS

### 2.1. Algorithms

The algorithms used for the searches, alignment, and homology estimates are described separately.

### 2.2. SSR search

The algorithm used for perfect and imperfect micro-/minisatellite searches was written in Perl and consists of the generation of a matrix that mixes A(adenine), T(thymine), C(cytosine), and G(guanine) in all possible composite arrangements between 1 and 10 nucleotides. The script instructions perform readings on fasta files, searching all possible arrangements in each database sequence.

Several instructions in the algorithm used in SSRLocator resemble those from MISA [16] and SSRIT [17]. However, additional instructions have been inserted in SSRLocator's code. Instead of allowing the overlap of a few nucleotides when two SSRs are adjacent to each other and one of them is shorter than the minimum size for a given class as found in MISA and SSRIT, a module written in Delphi language records the data and eliminates such overlaps.

The SSR Locator software contains windows focused on the selection and configuration of SSR and minisatellite types (mono- to 10-mers) and a minimum number of repeats for each one of the selected types. The algorithm calls a perfect repeat when one locus is present with adjacent loci at an up or downstream distance higher than 100 bp.

The algorithm calls an imperfect repeat when the same motif is present on both sides of a fragment containing up to 5 base pairs.

The algorithm identifies a composite locus when two or more adjacent loci were found at distances between 6 and 100 bp [16].

In this study, only “Class I” (≥20 bp) repeats are shown. These repeats have been described as the most efficient loci for use as molecular markers [17]. The software SSRLocator was configured to locate a minimum of 20 bp SSRs: monomers(x20), 2-mers(x10), 3-mers(x7), 4-mers(x5), 5-mers(x4), 6-mers(x4), and minissatellites: 7-mers(x3), 8-mers(x3), 9-mers(x3), and 10-mers(x3).

In order to validate the efficiency of SSRLocator in finding SSRs and minisatellites, the same database was analyzed withMISA and SSRIT, using the same parameters for minimum number of repeats.

### 2.3. Primer design

An algorithm written in Delphi language performs calls to Primer3 [19], which execute primer designs. These results are fed to a module that performs Virtual-PCRs and allocates individual identification, forward and reverse primer sequences, and a sequence fragment corresponding to the region flanked by the primers (original amplicon) to each SSR locus. A window allows the selection of Primer3 parameters, such as range of primer and amplicon sizes, as well as optimum primer size, ranges of melting temperature (TM) (minimum, maximum, and optimum) and GC content (minimum and optimum). For primer searches, the software automatically looks for five base pair distances from both SSR (5′ and 3′) flanking sites. In this study, the following parameters were used: amplicon size between 100 and 280 bp; minimum, optimum, and maximum annealing temperature (TM) of 45, 50, and 55, respectively, minimum, optimum, and maximum primer size of 15, 20, and 25 bp, respectively.

### 2.4. Virtual-PCR

The module used to simulate a PCR reaction was written in Delphi. The algorithm consists in reading the file generated by the previous module (SSR locus, forward and reverse primers, and original amplicon), followed by a search of sequences containing primer annealing sites. When annealing sites are found for the two primers, the flanked region and the primer sequences are copied to a new variable called “paralog amplicon.”

### 2.5. Global alignment

For the global alignment between paralog and original amplicon sequences and score calculations (match, mismatch, gaps), a routine was written in Delphi language using the algorithms of Needleman and Wunsch (1970) [20] and Smith and Waterman (1981) [21]. Also, in the same module, amplicon identities were calculated according to Waterman (1994) [22] and Vingron and Waterman (1994) [23].

### 2.6. Implementation

The strategy of creating a two-language hybrid program was established as a function of: (i) the higher speed achieved by handling large text files with Perl as compared to Delphi,and (ii) the better fitness of Perl for generating combinatory strings to be located. The Perl module was transformed into an executable file, making unnecessary to install Perl libraries during program installing. The graphic interface built, integrating input and output windows to the Windows operational system, was obtained using the Suite Turbo Delphi, where a menu system executes calls for each of the previously described modules.

### 2.7. Sequences for analysis

A total of 28 469 rice (*Oryza sativa* ssp. *japonica-* cv. Nipponbare) nonredundant full length nonredundant cDNA sequences, sequenced by *The Rice Full-Length cDNA Consortium*, mapped on the databases derived from the sequencing of *japonica* (*japonica* draft genome, BAC/PAC clones—IRGSP) and *indica* (*indica* draft genome) subspecies [24] were used for the analyses. These sequences are deposited in NCBI as two groups, the first comprising accesses from AK058203 to AK074028, and the second comprising accesses from AK98843 to AK111488. All these sequences can be also found in KOME (Knowledge-based Oryza Molecular Biological Encyclopedia).

**Figure 1:**
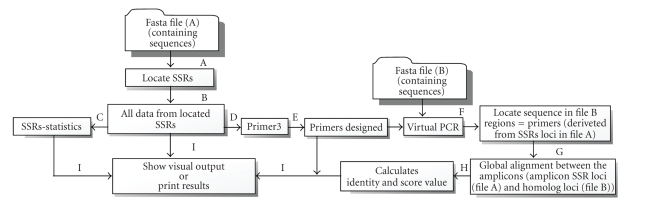
Flow-chart showing the functional structure of SSR Locator. (A) Perl script to search SSRs; (B) text file where information from detected SSRs is stored; (C) module for the statistical calculations for SSR motif occurrence; (D) module that formats text files into standard Primer3 input files; (E) running of Primer3; (F) module for running Virtual-PCR (using a second sequence file as a template); (G) module performing global alignment between homologous amplicons; (H) identity and alignment score calculations between homologous amplicons; and (I) file containing SSR, primer, homologous amplicons, identity, and score information.

A flow chart representing the different steps performed by the software is shown in Figure 1.

## 3. RESULTS

### 3.1. Program validation

A total of 3907 micro- and minisatellites were detected by SSRLocator in the 28 469 analyzed cDNA sequences. The same database searched with MISA and SSRIT presented 3913 and 3917 loci, respectively. The mono-, 4-mer, 6-mer, 7-mer, 8-mer, 9-mer, and 10-mer repeats were identical for the three programs. In the case of 2-mer repeats, 594 elements were detected by SSRLocator and 596 elements were detected by MISA and SSRIT. 3-mer repeats were differently scored by SSRLocator (1990) and the other two (1994) algorithms. For 5-mer repeats, SSRLocator and MISA found the same number of repeats (426), while SSRIT (430) found a different value.

### 3.2. Overall distribution of SSR types

The results obtained with SSRLocator indicate that out of 28 469 cDNA sequences, 3765 (13.22%) presented one or more micro-/minisatellite loci. In other studies, microsatellites were found in the following proportions in ESTs: 3% in arabidopsis [25], 4% in rosaceae [26], 8.11% in barley [16], 2.9% in sugarcane [27], and values ranging between 6–11% [28] and 1.5–4.7% [29] for cereals in general (maize, barley, rye, sorghum, rice, and wheat).

Considering the 3765 *fl*-cDNA sequences, in 3632 (92.96%) only a single micro-/minisatellitelocus was detected. In 125 sequences, two loci were detected, in seven sequences three lociandonly one sequence had four loci, adding up to 3907 occurrences. Among the types analyzed, SSRs (mono to 6-mer repeats) and minisatellites (7- to 10-mer repeats) comprised 96.98% and 4.12% of detected loci, respectively.

The distribution of occurrences detected by SSRLocator was consisted of 138 monomers, 594 2-mers, 1990 3-mers, 251 4-mers, 426 5-mers, 390 6-mers, 82 7-mers, 6 8-mers, 25 9-mers, and 5 10-mers, corresponding to rates of 3.53%, 15.20%, 50.93%, 6.42%, 10.90%, 9.98%, 2.10%, 0.15%, 0.64%, and 0.13%, respectively (see Table 1).

For the remaining SSRs, average percentage values have been reported as between 17 and 40% for 2-mer, 54–78% for 3-mer, 2.6–6.6% for 4-mer, 0.4–1.3% for 5-mer, and less than 1% for 6-mer repeats [28] and 26.5% for 2-mer, 65.4% 3-mer, 6.8% 4-mer, 0.77% 5-mer, and 0.45% for 6-mer repeats [30] for barley, maize, wheat, sorghum, rye, and rice, respectively. In nonredundant transcripts from the TIGR database, 15.6% 2-mer, 61.6% 3-mer, 8.5% 4-mer, and 14.4% 5-mer repeats were found in rice [31].

The frequency of micro/minisatellite locus occurrence for each million nucleotides (loci/Mb) [6] in this study was 2.94, 12.64, 42.34, 5.34, 9.06, 8.30, 1.74, 0.13, 0.53, and 0.11 for mono to 10-mer repeats/Mb, respectively. Overall occurrences of 83.13 loci/Mb were found (see Table 1). In other studies, different taxa were described in analyses of EST databases, such as 133 loci/Mb (barley), 161 loci/Mb (wheat, sorghum and rye), and 256 loci/Mb for rice [28]. Also, for nonredundant ESTs in rice, sorghum, barley, wheat, and Arabidopsis, frequencies of 277, 169, 112, 94 and 133 loci/Mb were found, respectively [30]. Frequencies closer to those found in this study were described for CDS regions of Rosaceaespecies, with an average of 40.9–78 loci/Mb for Rose, Almond and Peach, while 39 loci/Mb were found for Arabidopsis [26].

### 3.3. Occurrence patterns for different SSR and minisatellite types and motifs Monomers, 2-mers, 3-mers, and 4-mers

On Table 2, the contents and percentage values for different micro-/minisatellite motifs are shown. For monomer, 2-mer and 3-mer repeats, all possible arrangements are shown, while for 4-mer to 10-mer repeats, only the ten most frequent motifs are shown.

The A/T monomer repeats were found in 125 loci, with 111 (88.80%) and 14 (11.20%) loci formed by A and T nucleotides, respectively. The C/G motifs were found in 13 loci, with ten (76.92%) and three (23.08%) loci formed by C and G, respectively. A/T containing SSRs were predominant and comprised 90.58% of monomer loci. In the overall distribution, the monomers represent 3.53% of 3907 detected loci. Motifs AG/CT and GA/TC were the most frequent and added up to 8.52% of 2-mer SSRs, and 6.89% and 5.96% of all 3907 detected occurrences. The motifs CT, GA, and TC were the most abundant adding up to 172, 143, and 90 loci, respectively. In maize, barley, rice, sorghum, and wheat ESTs, the motif AG was described as the most frequent [6, 16, 28, 29, 31, 32]. However, in some studies, the most frequent motif was GA [30, 33]. Repeats composed by guanine and cytosine were the most abundant among trimers, with occurrences of 18.44%, 17.89%, and 10.60%, respectively, for the motifs CCG/CGG, CGC/GCG, and GCC/GGC, adding up to 23.9% of the overall frequencies of micro-/minisatellites in the analysis. The motifs CGC, CCG, and CGG were the most frequent comprising 218, 197, and 170 loci, respectively. Many reports indicate the 3-mer CCG as the most frequent in maize, barley, wheat, sorghum and rye [6, 16, 28, 32], sugarcane [27] and rice [29, 31].

Among 4-mers, 100 different arrangements were found, where the motifs GATC (7.17%), ATTA/AAT (6.77%), and ATCG/CGAT (5.98%) were the most frequent. These motifs add up to 19.92% of 4-mer repeats found and represent 1.28% of the overall content of micro-/minisatellites. In barley ESTs, ACGT was reported as the most abundant motif [16, 28]. For other species, AAAG/CTTT and AAGG/CCTT in *Lolium perene* [34], AAAG/CTTT and AAAC/GTTT in arabidopsis UTRs [6, 35], and AAAT and AAAG in citrus [36, 37] were described as most abundant.

### 3.4. Remaining repeats

Among 5-mers, 188 different arrangements were detected and the most frequent were CTCCT, CTCTC, and CCTCC with 17, 17, and 12 occurrences, respectively. In the analysis of CDS regions, the ACCCG motif was the most frequent in Arabidopsis, AAAAG in *S. cerevisae*, *C. elegans*, and AAAAC in different primates [38]. Also, the motifs AAAAT, AAAAC, and AAAAG were described as the most frequent in eukaryotes [39]. In rice, the motifs AGAGG and AGGGG were the most abundant [31]. Repeats of type 6-mer were detected in 230 different arrangements, where CGCCTC and TCGCCG were the most frequent, occurring in 12 and 10 loci, respectively. Other studies have shown higher frequencies for the motifs AAGATG, AAAAAT in arabidopsis [35], AAAAAG in citrus [36], AACACG in *S. cerevisae*, ACCAGG in *C. elegans* and CCCCGG in primates [38]. For all remaining repeats (minisatellites), the occurrences are widely distributed with low-percentage values for each arrangement. For 7-mer, 8-mer, 9-mer, and 10-mer repeats, the totals of occurrences were 57, 5, 23, and 5, respectively.

### 3.5. Primer design and PCR simulation

The design of primers for the 3907 detected micro-/minisatellites resulted in 3329 primer pairs, covering 85.20% of loci. The running of “Virtual PCR” generated a total of 4610 amplicons. A module in SSRLocator checks for primer redundancy. A total of 2397 primer pairs amplified only the fragment from its original locus (specific amplicons) and 932 pairs amplified one or more regions besides the original locus. From these, 692 pairs amplified two fragments, one from the original site and a second from another region (paralogous). In this case, 692 specific amplicons plus 692 redundant amplicons, were detected. A total of 143, 90, 2, and 5 primer pairs generated three (two redundancies), four (three redundancies), five (four redundancies), and six (five redundancies) fragments, respectively. The final product of 932 primers with more than one anchoring region resulted in 932 specific amplicons and 1281 redundant amplicons, adding up to 2213 fragments.

To investigate the ability of these primers in amplifying genomic sequences, an extra experiment was performed against the whole rice genomic sequence available at NCBI. The different groups of redundant and nonredundant primer sets, that is, amplifying one, two, three, or more times in the cDNA database, were tested against the genomic sequence. From the 2397 nonredundant primers, only 924 amplified a locus in the genomic sequence. This difference was already expected because of difficulties in amplifying genomic regions, that is, if some primers anneal to a boundary region between two exons in the cDNA, the presence of introns would make this annealing site no more available. It is interesting to note that from the 924 amplicons detected, 914 (99%) did amplify only one locus in the genomic region, agreeing with the cDNA results. When the primer sets that amplified two different cDNAs were run against the genomic sequence, only 294/692 (42.5%) did amplify, having 14.5% been able to amplify two different loci. Only one primer set did amplify more than two loci. These results indicate that SSR locator performance was consistent between the two databases regarding the nonredundant loci, that is, from those loci that were able to be amplified in both databases, their status of nonredundant was maintained. The changes observed for the redundant loci can be attributable to many causes, including redundancy in the cDNA database, but also to biological reasons due to primer positioning.

### 3.6. Identity between specific and redundant amplicons

Results of a global alignment between amplicons from original and redundant sites are shown in Table 3. Among the 1281 redundant amplifications, 787 (61.44%) resulted in a perfect alignment between both loci (identity equal to 100). For redundant amplicons with identity levels of 96–99%, and 90–95%, 452 (35.28%) and 8 (0.62%) loci were found, respectively. Alignments with identity levels bellow 90% were found in only 2.65% of cases. The fact that such a high percentage of redundant loci show high identity is probably a consequence of the genome fraction chosen, that is, expressed sequences. This fraction is under tight selection pressure and should not accumulate variations such as substitutions or indels at a high rate. As expected, comparisons to whole genome, generated a great deal of polymorphism, due to the inclusion of intronic regions in the alignments (data not shown).

## 4. CONCLUSIONS

The software SSRLocator was successfully implemented, adding steps for (1) SSR discovery, (2) primer design, and (3) PCR simulation between the primers obtained from original sequences and other fasta files. Also, the software produces reports for frequency of occurrence, nucleotide arrangement, primer lists with all standard information needed for PCR and global alignments. From the PCR simulation, it was possible to point out which primer pairs were nonredundant, suggesting that these primers are more appropriate for mapping purposes. In this case, however, wet lab experiments should be performed to confirm the advantage of nonredundant over redundant primers for mapping.

It is possible that the results for micro-/minisatellite frequencies (loci/Mb) obtained in this study diverge from the results found in the literature. This can be explained by the different databases used (redundant ESTs, nonredundant ESTs and/or fl-cDNA), different algorithm configurations and minimum requirements set for counting motifs. Another explanation for some contrasting results is the fact that only “Class I” repeats were analyzed in our study.

The results showed that 932 (27.99%) primers presented amplifications in more than one gene sequence. This could be mostly due to the fact that primer pairs derived from a specific gene (cDNA) anchored in similar sites in other duplicated genes, since 5,607/28,469 (19.70%) genes were described as paralogs in the annotation of the database used [24]. Gene duplication along with polyploidy and transposon amplification are the major driving forces in genome evolution [40]. It is therefore not surprising that so many loci have redundancy. Also, a second possibility is that some primers were generated from protein domain regions within the analyzed cDNAs. These domains could be found in protein families with many genome copies, resulting in the observed redundancies. A validation of the redundancies of cDNA results was obtained through a virtual-PCR against the whole rice genome sequence. From the nonredundant primers that generated an amplicon, ca. 99% were nonredundant.

Finally, this tool can be used successfully for data mining strategies to find SSR primers in genomic or expressed sequences (ESTs/cDNAs). Also, this software can be a tool for microsatellite discovery in databanks of related species, anchoring primers in ortholog or paralog regions contained between databases from two different species.

## Figures and Tables

**Table 1:** Distribution of SSR/minisatellite motifs according to the number of repeats.

Repeats	**Mono-**	(%)	**2-mer**	(%)	**3-mer**	(%)	**4-mer**	(%)	**5-mer**	(%)	**6-mer**	(%)	**7-mer**	(%)	**8-mer**	(%)	**9-mer**	(%)	**10-mer**	(%)	**Total**	(%)
3	**0**	—	**0**	—	**0**	—	**0**	—	**0**	—	**0**	—	**78**	95.12	**6**	100	**24**	96	**5**	100	**113**	2.89
4	**0**	—	**0**	—	**0**	—	**0**	—	**348**	81.69	**323**	82.82	**4**	4.88	**0**	0	**1**	4	**0**	0	**676**	17.30
5	**0**	—	**0**	—	**0**	—	**181**	72.11	**69**	16.20	**45**	11.54	**0**	0	**0**	0	**0**	0	**0**	0	**295**	7.55
6	**0**	—	**0**	—	**0**	—	**41**	16.33	**7**	1.64	**13**	3.33	**0**	0	**0**	0	**0**	0	**0**	0	**61**	1.56
7	**0**	—	**0**	—	**1220**	61.31	**9**	3.59	**0**	0	**5**	1.28	**0**	0	**0**	0	**0**	0	**0**	0	**1234**	31.58
8	**0**	—	**0**	**—**	**441**	22.16	**9**	3.59	**1**	0.23	**1**	0.26	**0**	0	**0**	0	**0**	0	**0**	0	**452**	11.57
9	**0**	—	**0**	—	**173**	8.69	**4**	1.59	**0**	0	**1**	0.26	**0**	0	**0**	0	**0**	0	**0**	0	**178**	4.56
10	**0**	—	**125**	21.04	**68**	3.42	**1**	0.40	**0**	0	**2**	0.51	**0**	0	**0**	0	**0**	0	**0**	0	**196**	5.02
11	**0**	—	**82**	13.80	**32**	1.61	**3**	1.20	**0**	0	**0**	0	**0**	0	**0**	0	**0**	0	**0**	0	**117**	2.99
12	**0**	—	**76**	12.79	**18**	0.90	**1**	0.40	**0**	0	**0**	0	**0**	0	**0**	0	**0**	0	**0**	0	**95**	2.43
13	**0**	—	**71**	11.95	**5**	0.25	**1**	0.40	**0**	0	**0**	0	**0**	0	**0**	0	**0**	0	**0**	0	**77**	1.97
14	**0**	—	**39**	6.57	**2**	0.10	**0**	0	**0**	0	**0**	0	**0**	0	**0**	0	**0**	0	**0**	0	**41**	1.05
15	**0**	—	**44**	7.41	**5**	0.25	**0**	0	**1**	0.23	**0**	0	**0**	0	**0**	0	**0**	0	**0**	0	**50**	1.28
16	**0**	—	**30**	5.05	**2**	0.10	**0**	0	**0**	0	**0**	0	**0**	0	**0**	0	**0**	0	**0**	0	**32**	0.82
17	**0**	—	**33**	5.56	**1**	0.05	**0**	0	**0**	0	**0**	0	**0**	0	**0**	0	**0**	0	**0**	0	**34**	0.87
18	**0**	—	**15**	2.53	**3**	0.15	**0**	0	**0**	0	**0**	0	**0**	0	**0**	0	**0**	0	**0**	0	**18**	0.46
19	**0**	—	**17**	2.86	**1**	0.05	**1**	0	**0**	0	**0**	0	**0**	0	**0**	0	**0**	0	**0**	0	**19**	0.49
20	**21**	15.22	**14**	2.36	**2**	0.10	**0**	0	**0**	0	**0**	0	**0**	0	**0**	0	**0**	0	**0**	0	**37**	0.95
21	**19**	13.77	**8**	1.35	**2**	0.10	**0**	0	**0**	0	**0**	0	**0**	0	**0**	0	**0**	0	**0**	0	**29**	0.74
22	**15**	10.87	**6**	1.01	**3**	0.15	**0**	0	**0**	0	**0**	0	**0**	0	**0**	0	**0**	0	**0**	0	**24**	0.61
23	**8**	5.80	**7**	1.18	**3**	0.15	**0**	0	**0**	0	**0**	0	**0**	0	**0**	0	**0**	0	**0**	0	**18**	0.46
24	**3**	2.17	**5**	0.84	**0**	0	**0**	0	**0**	0	**0**	0	**0**	0	**0**	0	**0**	0	**0**	0	**8**	0.20
25	**9**	6.52	**5**	0.84	**1**	0.05	**0**	0	**0**	0	**0**	0	**0**	0	**0**	0	**0**	0	**0**	0	**15**	0.38
26	**5**	3.62	**4**	0.67	**2**	0.10	**0**	0	**0**	0	**0**	0	**0**	0	**0**	0	**0**	0	**0**	0	**11**	0.28
27	**3**	2.17	**1**	0.17	**1**	0.05	**0**	0	**0**	0	**0**	0	**0**	0	**0**	0	**0**	0	**0**	0	**5**	0.13
28	**1**	0.72	**3**	0.51	**3**	0.15	**0**	0	**0**	0	**0**	0	**0**	0	**0**	0	**0**	0	**0**	0	**7**	0.18
29	**4**	2.90	**0**	0	**1**	0.05	**0**	0	**0**	0	**0**	0	**0**	0	**0**	0	**0**	0	**0**	0	**5**	0.13
30	**2**	1.45	**0**	0	**0**	0	**0**	0	**0**	0	**0**	0	**0**	0	**0**	0	**0**	0	**0**	0	**2**	0.05
31	**9**	6.52	**2**	0.34	**1**	0.05	**0**	0	**0**	0	**0**	0	**0**	0	**0**	0	**0**	0	**0**	0	**12**	0.31
32	**3**	2.17	**3**	0.51	**0**	0	**0**	0	**0**	0	**0**	0	**0**	0	**0**	0	**0**	0	**0**	0	**6**	0.15
33	**3**	2.17	**0**	0	**0**	0	**0**	0	**0**	0	**0**	0	**0**	0	**0**	0	**0**	0	**0**	0	**3**	0.08
34	**1**	0.72	**1**	0.17	**0**	0	**0**	0	**0**	0	**0**	0	**0**	0	**0**	0	**0**	0	**0**	0	**2**	0.05
35	**6**	4.35	**1**	0.17	**0**	0	**0**	0	**0**	0	**0**	0	**0**	0	**0**	0	**0**	0	**0**	0	**7**	0.18
36	**1**	0.72	**1**	0.17	**0**	0	**0**	0	**0**	0	**0**	0	**0**	0	**0**	0	**0**	0	**0**	0	**2**	0.05
37	**1**	0.72	**0**	0	**0**	0	**0**	0	**0**	0	**0**	0	**0**	0	**0**	0	**0**	0	**0**	0	**1**	0.03
38	**4**	2.90	**0**	0	**0**	0	**0**	0	**0**	0	**0**	0	**0**	0	**0**	0	**0**	0	**0**	0	**4**	0.10
39	**0**	0	**1**	0.17	**0**	0	**0**	0	**0**	0	**0**	0	**0**	0	**0**	0	**0**	0	**0**	0	**1**	0.03
40	**1**	0.72	**0**	0	**0**	0	**0**	0	**0**	0	**0**	0	**0**	0	**0**	0	**0**	0	**0**	0	**1**	0.03
41	**1**	0.72	**0**	0	**0**	0	**0**	0	**0**	0	**0**	0	**0**	0	**0**	0	**0**	0	**0**	0	**1**	0.03
42	**2**	1.45	**0**	0	**0**	0	**0**	0	**0**	0	**0**	0	**0**	0	**0**	0	**0**	0	**0**	0	**2**	0.05
43	**2**	1.45	**0**	0	**0**	0	**0**	0	**0**	0	**0**	0	**0**	0	**0**	0	**0**	0	**0**	0	**2**	0.05
44	**0**	0	**0**	0	**0**	0	**0**	0	**0**	0	**0**	0	**0**	0	**0**	0	**0**	0	**0**	0	**0**	0.00
≥ 45	**14**	10.14	**0**	0	**0**	0	**0**	0	**0**	0	**0**	0	**0**	0	**0**	0	**0**	0	**0**	0	**14**	0.36

Total	**138**		**594**		**1990**		**251**		**426**		**390**		**82**		**6**		**25**		**5**		**3907**	
(%)	**3.53**		**15.20**		**50.93**		**6.42**		**10.90**		**9.98**		**2.10**		**0.15**		**0.64**		**0.13**		**100.00**	

**Table 2:** Distribution of SSR/minisatellite repeats in the rice cDNA collection.

Motif		Ocur^(1)^	(%)^(1)^	Ocur^(2)^	(%)^(2)^	Total	(%) Group	(%) Overall
Mono-	A/T	**111**	88.80	**14**	11.20	**125**	90.58	3.20
	C/G	**10**	76.92	**3**	23.08	**13**	9.42	0.33

2-mer	AG/CT	**97**	36.06	**172**	63.94	**269**	45.29	6.89
	GA/TC	**143**	61.37	**90**	38.63	**233**	39.23	5.96
	CA/TG	**10**	35.71	**18**	64.29	**28**	4.71	0.72
	AT	**24**	100.00	**—**	—	**24**	4.04	0.61
	AC/GT	**6**	31.58	**13**	68.42	**19**	3.20	0.49
	TA	**19**	100.00	**—**	—	**19**	3.20	0.49
	CG	**2**	100.00	**—**	—	**2**	0.34	0.05

3-mer	CCG/CGG	**197**	53.68	**170**	46.32	**367**	18.44	9.39
	CGC/GCG	**218**	61.24	**138**	38.76	**356**	17.89	9.11
	GCC/GGC	**112**	53.08	**99**	46.92	**211**	10.60	5.40
	CTC/GAG	**73**	42.69	**98**	57.31	**171**	8.59	4.38
	AGG/CCT	**34**	30.91	**76**	69.09	**110**	5.53	2.82
	GGA/TCC	**60**	62.50	**36**	37.50	**96**	4.82	2.46
	CAG/CTG	**58**	76.32	**18**	23.68	**76**	3.82	1.95
	AAG/CTT	**34**	50.75	**33**	49.25	**67**	3.37	1.71
	CGA/TCG	**33**	54.10	**28**	45.90	**61**	3.07	1.56
	AGC/GCT	**36**	62.07	**22**	37.93	**58**	2.91	1.48
	GCA/TGC	**47**	83.93	**9**	16.07	**56**	2.81	1.43
	AGA/TCT	**33**	62.26	**20**	37.74	**53**	2.66	1.36
	CCA/TGG	**39**	75.00	**13**	25.00	**52**	2.61	1.33
	ACC/GGT	**22**	48.89	**23**	51.11	**45**	2.26	1.15
	GAA/TTC	**28**	63.64	**16**	36.36	**44**	2.21	1.13
	CAC/GTG	**28**	65.12	**15**	34.88	**43**	2.16	1.10
	GAC/GTC	**18**	54.55	**15**	45.45	**33**	1.66	0.84
	ACG/CGT	**11**	42.31	**15**	57.69	**26**	1.31	0.67
	ATC/GAT	**5**	45.45	**6**	54.55	**11**	0.55	0.28
	TCA/TGA	**5**	50.00	**5**	50.00	**10**	0.50	0.26
	CAA/TTG	**4**	50.00	**4**	50.00	**8**	0.40	0.20
	ACT/AGT	**3**	42.86	**4**	57.14	**7**	0.35	0.18
	TAA/TTA	**1**	14.29	**6**	85.71	**7**	0.35	0.18
	CTA/TAG	**4**	66.67	**2**	33.33	**6**	0.30	0.15
	AAT/ATT	**1**	20.00	**4**	80.00	**5**	0.25	0.13
	CAT/ATG	**4**	100.00	**0**	0	**4**	0.20	0.10
	AAC/GTT	**3**	75.00	**1**	25.00	**4**	0.20	0.10
	ATA/TAT	**1**	50.00	**1**	50.00	**2**	0.10	0.05
	GTA/TAC	**1**	100.00	**0**	0	**1**	0.05	0.03

4-mer	GATC	**18**	100.00	**0**	0	**18**	7.17	0.46
	ATTA/TAAT	**9**	52.94	**8**	47.06	**17**	6.77	0.44
	ATCG/CGAT	**3**	20.00	**12**	80.00	**15**	5.98	0.38
	CATC/GATG	**4**	40.00	**6**	60.00	**10**	3.98	0.26
	AGAA/TTCT	**2**	25.00	**6**	75.00	**8**	3.19	0.20
	GCTA/TAGC	**6**	75.00	**2**	25.00	**8**	3.19	0.20
	GATA/TATC	**1**	14.29	**6**	85.71	**7**	2.79	0.18
	GCGA/TCGC	**3**	42.86	**4**	57.14	**7**	2.79	0.18
	GCAC/GTGC	**2**	33.33	**4**	66.67	**6**	2.39	0.15
	AGGG/CCCT	**2**	33.33	**4**	66.67	**6**	2.39	0.15

5-mer	AGGAG/CTCCT	**3**	15.00	**17**	85.00	**20**	4.69	**0.51**
	CTCTC/GAGAG	**17**	89.47	**2**	10.53	**19**	4.46	**0.49**
	GAGGA/TCCTC	**9**	56.25	**7**	43.75	**16**	3.76	**0.41**
	CCTCC/GGAGG	**12**	80.00	**3**	20.00	**15**	3.52	**0.38**
	AGAGG/CCTCT	**4**	26.67	**11**	73.33	**15**	3.52	**0.38**
	GGAGA/TCTCC	**2**	18.18	**9**	81.82	**11**	2.58	**0.28**
	CTCGC/GCGAG	**7**	77.78	**2**	22.22	**9**	2.11	**0.23**
	AGCTA/TAGCT	**4**	44.44	**5**	55.56	**9**	2.11	**0.23**
	GAAAA/TTTTC	**2**	25.00	**6**	75.00	**8**	1.88	**0.20**
	AGGCG/CGCCT	**2**	25.00	**6**	75.00	**8**	1.88	**0.20**

6-mer	CGCCTC/GAGGCG	**12**	85.71	**2**	14.29	**14**	3.59	0.36
	CGGCGA/TCGCCG	**4**	28.57	**10**	71.43	**14**	3.59	0.36
	CCTCCG/CGGAGG	**9**	81.82	**2**	18.18	**11**	2.82	0.28
	AGGCGG/CCGCCT	**1**	10.00	**9**	90.00	**10**	2.56	0.26
	CCGTCG/CGACGG	**4**	44.44	**5**	55.56	**9**	2.31	0.23
	CGTCGC/GCGACG	**7**	77.78	**2**	22.22	**9**	2.31	0.23
	ACCGCC/GGCGGT	**1**	12.50	**7**	87.50	**8**	2.05	0.20
	CCACCG/CGGTGG	**6**	85.71	**1**	14.29	**7**	1.79	0.18
	GGCGGA/TCCGCC	**5**	71.43	**2**	28.57	**7**	1.79	0.18
	CTCCAT/ATGGAG	**6**	100.00	**0**	0	**6**	1.54	0.15

7-mer	CCGCCGC/GCGGCGG	**4**	66.67	**2**	33.33	**6**	7.32	0.15
	CTCTCTC/GAGAGAG	**4**	80.00	**1**	20.00	**5**	6.10	0.13
	CCTCTCT/AGAGAGG	**4**	100.00	**0**	0	**4**	4.88	0.10
	CTCTCTT/AAGAGAG	**4**	100.00	**0**	0	**4**	4.88	0.10
	CCCAAAT/ATTTGGG	**3**	100.00	**0**	0	**3**	3.66	0.08
	GCCGCCG/CGGCGGC	**3**	100.00	**0**	0	**3**	3.66	0.08
	GCGGCGC/GCGCCGC	**2**	100.00	**0**	0	**2**	2.44	0.05
	AATAAAA/TTTTATT	**2**	100.00	**0**	0	**2**	2.44	0.05
	GTGTGCG/CGCACAC	**2**	100.00	**0**	0	**2**	2.44	0.05
	CGCCGTC/GACGGCG	**2**	100.00	**0**	0	**2**	2.44	0.05

8-mer	TTGGTTTC/GAAACCAA	**2**	100.00	**0**	0	**2**	33.33	0.05
	TGGGCTTG/CAAGCCCA	**1**	100.00	**0**	0	**1**	16.67	0.03
	GCTTCTTG/CAAGAAGC	**1**	100.00	**0**	0	**1**	16.67	0.03
	ACGGGCGA/TCGCCCGT	**1**	100.00	**0**	0	**1**	16.67	0.03
	ATGATGTA/TACATCAT	**1**	100.00	**0**	0	**1**	16.67	0.03

9-mer	TCGGCGGCG/CGCCGCCGA	**2**	100.00	**0**	0	**2**	8.00	0.05
	AGGTGGTGG/CCACCACCT	**2**	100.00	**0**	0	**2**	8.00	0.05
	CCGGTGCGA/TCGCACCGG	**1**	100.00	**0**	0	**1**	4.00	0.03
	ACGAGGAGG/CCTCCTCGT	**1**	100.00	**0**	0	**1**	4.00	0.03
	TCCCTTTTC/GAAAAGGGA	**1**	100.00	**0**	0	**1**	4.00	0.03
	CGGCATGAA/TTCATGCCG	**1**	100.00	**0**	0	**1**	4.00	0.03
	CGGCAGCGA/TCGCTGCCG	**1**	100.00	**0**	0	**1**	4.00	0.03
	ACCATCCCG/CGGGATGGT	**1**	100.00	**0**	0	**1**	4.00	0.03
	ATGGGCGGC/GCCGCCCAT	**1**	100.00	**0**	0	**1**	4.00	0.03
	ATGCAGGGT/ACCCTGCAT	**1**	100.00	**0**	0	**1**	4.00	0.03

10-mer	AGCCCCAACG/CGTTGGGGCT	**1**	50.00	**1**	50.00	**2**	40.00	0.05
	TTTTTTTCTT/AAGAAAAAAA	**1**	100.00	**0**	0	**1**	20.00	0.03
	CCTGCTTTGC/GCAAAGCAGG	1	100	0	0	1	20	0.03
	ATCTCCGCCG/CGGCGGAGAT	1	100	0	0	1	20	0.03

**Table 3:** Distribution of amplicon alignments for specific and redundant amplicons with varying identity levels.

Identity	100	99	98	97	96	95–90	89–80	79–70	69–60	≤59	Total
Amplicons	787	261	151	29	11	8	8	6	5	15	1281
%	61.44	20.37	11.79	2.26	0.86	0.62	0.62	0.47	0.39	1.17	—
